# Identifying reasons for non-acceptance of influenza vaccine in healthcare workers: an observational study using declination form data

**DOI:** 10.1186/s12913-023-10141-2

**Published:** 2023-10-27

**Authors:** Aimee Challenger, Petroc Sumner, Eryl Powell, Lewis Bott

**Affiliations:** 1 World Health Organization Collaborating Centre On Investment for Health and Wellbeing, Public Health Wales, Cardiff, Wales UK; 2 School of Psychology, Cardiff University, Tower Building, 70 Park Place, Cardiff, CF10 3AT Wales UK; 3 Aneurin Bevan Gwent Public Health Team, Aneurin Bevan University Health Board, Wales, UK

**Keywords:** Vaccination, Influenza, Declination forms, Attitudes, Risk perception

## Abstract

**Background:**

Healthcare workers are sometimes required to complete a declination form if they choose not to accept the influenza vaccine. We analysed the declination data with the goal of identifying barriers to vaccination uptake across seasons, staff groups, and pre- and post- arrival of COVID-19.

**Methods:**

Reasons for declining the vaccine were gathered from *N* = 2230 declination forms, collected over four influenza seasons, 2017/2018, 2018/2019, 2019/2020 and 2020/2021, from a single health board in the UK. Reasons were classified according to ten categories and the resulting distribution analysed across year and staff groups. A further analysis considered the two most prevalent categories in more detail.

**Results:**

*Fear of adverse reactions* and *Lack of perception of own risk* were identified as primary reasons for not accepting the vaccine across time and across staff groups. However, there was no evidence that *Lack of concern* with influenza, or *Doubts about vaccine efficacy* was prevalent, contrary to previous findings. Overall, reasons fitted a pattern of underestimating risk associated with influenza and overestimating risk of minor adverse reactions. There were also differences across years, χ^2^(24) = 123, *p* < .001. In particular, there were relatively fewer *Lack of perception of own risk* responses post-COVID-19 arrival than before, χ^2^(8) = 28.93, *p* = .002.

**Conclusion:**

This study shows that data collected from declination forms yields sensible information concerning vaccine non-acceptance without the difficulties of retrospective or pre-emptive reasoning suffered by questionnaires. Our findings will aid messaging campaigns designed to encourage uptake of the influenza vaccine in healthcare workers. In particular, we argue for an approach focused on risk perception rather than correction of straightforward misconceptions.

Seasonal influenza is responsible for a substantial number of deaths worldwide. As of 2017, global influenza-associated respiratory deaths were estimated to be 290,000 – 645,000 per annum [1]. In addition, seasonal influenza increases winter pressures on health systems. For example, in Wales, a UK nation of 3.1 million, there were 904 influenza confirmed admissions to A&E, 694 hospitalizations and 59 patients in intensive care units in 2019/2020 [2]. There were also 26 outbreaks in care environments.

Influenza outbreaks in hospitals and other care environments can have more serious consequences than elsewhere because of the elevated proportion of elderly and immunocompromised patients. It is therefore important that those who care for these individuals are vaccinated, both for their own safety and to provide indirect protection for patients. The vaccine is a safe and effective intervention to prevent both influenza and its secondary complications [3–5], and to reduce illness-related absence in healthcare workers (HCW) [6]. However, while vaccinations targets are typically high, they are notoriously difficult to attain [7]. For example, uptake amongst frontline HCW in Wales 2019/2020 was 59% [2]—far below the 75% WHO recommendation [8] and Welsh Government target. There is also considerable variation within HCW staff groups. In Wales 2019/2020 [2], for example, 63% of Medical and Dental staff were vaccinated, but only 40% of Estates staff.

In this study, we seek to identify attitudes that prevent frontline HCW from accepting the vaccine. While other work has sought the same information [9–11], we adopted a novel technique of analysing responses from vaccine declination forms (“no thank you” forms).

## Attitudes preventing uptake

There are many structural barriers to vaccination [7] but there are also attitudes towards the vaccine that prevent uptake [9–18]. For example, Hollmeyer et al. [14] conducted a widely cited review of 25 studies examining self-reported reasons for influenza vaccine acceptance or refusal amongst HCW. They found that the most cited reason for vaccine refusal was *Fear of adverse reactions*. This was followed by *Lack of concern* [for the seriousness of influenza], *Inconvenient delivery*, *Lack of perception of own risk,* and *Doubts about vaccine efficacy*. More recent studies are consistent with Hollmeyer’s review [10, 18, 19]. For example, Ferragut et al. [10] conducted a questionnaire study with HCW (*N* = 94) who worked within the clinical area of an acute London hospital. The most common reason for vaccine refusal was, “I got sick after the vaccine” (*N* = 37, 39%), i.e. *Fear of adverse reactions*.

Questionnaire-based studies like those above are an important source of information about why people decline the vaccine. Nonetheless, questionnaires are imperfect tools for acquiring information about vaccine behaviour. Responses are either retrospective (“Why did I decline the vaccine?”), or pre-emptive and counterfactual (“if I were to be offered the vaccine, what would I say?”), and so may not reflect declination reasons (or even decisions) when they are offered the vaccine. Furthermore, most existing studies reflect attitudes prior to the COVID-19 pandemic and reasoning about vaccine acceptance may have subsequently changed [20–23].

## Declination forms

One approach to increasing uptake is to introduce declination forms if HCW decline the vaccine [24–26] For example, in 2018/2019, health trusts in England required decliners to sign a statement declaring their knowledge of the health consequences of not being vaccinated, and to state reasons for refusal from a specified list [27, 28]. The reasoning behind declination forms is that healthcare workers who are undecided or have neutral views of the influenza vaccination may be persuaded to accept it if they cannot identify good reasons for non-acceptance [7].

Although evidence that declination forms increase uptake is weak [29], an uncontested benefit is that they provide informative data on why HCW decline the vaccine. Declination data is less sensitive to sampling bias than other methods since every participant who declines a vaccine is asked to complete a declination form (although not all forms are completed). Data is also collected at the point of refusal, thereby obviating the need for participants to retrospectively recall their reasons for declining or to pre-empt what their reasons might be.

Surprisingly, there is only one study [30] that reports reasons given on declination forms. Ribner et al. [30] describe the implementation of a mandatory declination form policy in one healthcare system in the USA, 2006–2007. The main focus of the paper was on the effects of declination forms on uptake, and there were no comparisons with other studies investigating reasons for declination. Nonetheless, the primary declination reasons described were broadly consistent with questionnaire studies: *Afraid of side effects* (28%); *I never get the flu* (25%), and *Fear of getting influenza from the vaccine* (19%). Reasons for declination may have changed since 2006–2007, however, and may vary according to location and policy implementation. Ribner et al. [30] also used pre-determined response options rather than free responses and so there is only limited detail available about the declination. We therefore sought to analyse declination data from more recent sources and to establish whether these findings generalise to other contexts.

We analysed declination data collected from one health board in Wales, UK, Aneurin Bevan University Health Board (ABUHB). The data set spans four years, 2017/2018, 2018/2019, 2019/2020, 2020/2021, three of which were prior to the arrival of the COVID-19 pandemic, one of which was post-arrival (although prior to the wide-spread roll out of COVID-19 vaccines in the UK). Declination responses were categorised using an extended version of the nine category model described by Hollmeyer et al. [14]. We used the Hollmeyer et al. model because using the same model facilitated cross-study and cross-time comparisons. There were two primary aims of the study. First, to understand whether the declination reasons given by HCW have changed across years, in particular pre- and post- COVID arrival. Second, to establish whether declination reasons change across staff groups.

The primary analysis suggested interesting departures from the literature with respect to myths and misconceptions around vaccines. Further analysis was therefore conducted at the subcategory level for *Fear of adverse reactions* and *Lack of perception of own risk*, two of the most prevalent declination categories. We tested (1) whether responses were consistent with medically described adverse reactions [31] i.e., a small increased risk of fever and muscle ache (myalgia) (2) whether participants believed that the vaccine caused influenza, a myth reported in many studies [11, 13, 27, 30], and (3) an analysis of what determined a participant’s perception of risk of contracting influenza. The subcategory analysis consisted of testing the occurrence of strings associated with the research questions e.g., whether “fever” occurred frequently in the *Fear of adverse reactions* category.

## Method

### Ethical approval

The study was considered Service Evaluation by ABUHB Research and Development Department, Research Risk Review Panel, SA/1181/20 and participant consent was waived.

### Data storage

Raw data is publicly available at https://osf.io/734tg/?view_only=77213145821449cd9008980ef383b243

### Environment

Data was drawn from declination forms collected by occupational health and flu champion staff at ABUHB. ABUHB was established on the 1st October 2009 and covers the areas of Blaenau Gwent, Caerphilly, Monmouthshire, Newport, Torfaen and South Powys in Wales, UK. The Health Board employs approximately 14,000 staff, two thirds of whom are involved in direct patient care. There are more than 250 consultants in a total of over 1000 hospital and general practice doctors, 6,000 nurses, midwives, allied professionals and community workers.

ABUHB offered influenza vaccinations via the Occupational Health department and the “Flu Champions” system. The vaccine was offered at staff meetings, training sessions and conferences, and via “walk up” sessions in high footfall areas such as canteens. Flu champions vaccinated their peers on wards or in teams. Staff numbers, targets, and vaccination uptake rates are shown in Table 1.

**Table 1:** ABUHB descriptors

	Influenza season			
	2017/18	2018/19	2019/20	2020/21
Number of frontline ABUHB staff	9577	8901	9133	9537
Welsh Government targets for frontline staff	60%	60%	65%	75%
ABUHB uptake rate for frontline staff	58.0%	62.4%	61.9%	67.0%
NHS Wales uptake rate for frontline staff	57.9%	55.5%	58.9%	65.6%

Data was recorded electronically by immunisers via an “Inactivated influenza vaccine no thank you form”. Data was collected during four influenza seasons 2017/2018, 2018/2019, 2019/2020, 2020/2021. Declination forms were anonymous and collected the following information about staff members: Gender, Job title, Division, Reason for refusal. Once staff members had given the above information, immunisers were instructed to refer to the back of the declination form where a list of common responses and misconceptions about the influenza vaccination were listed. This information was present to encourage staff who may have been misinformed about the influenza vaccination. Immunisers recorded whether the member of staff changed their mind (of which 23 decliners did so), along with the immuniser’s name, date, time, and signature (anonymised for analysis).

HCW were not required to sign or give their name.

### Design

#### Independent variables

Staff group and year were used as independent variables. Staff groups were those used by the NHS when reporting the number of directly employed NHS staff [31]. These were Medical and dental staff; Nursing, midwifery and health visiting; Scientific, therapeutic and technical; Administration and estates; Healthcare assistants and other support workers; Other; and Unknown. Counts of declination forms by staff group and year are shown in Table 2.

**Table 2:** Number of declination forms by staff group and year

	2017/2018	2018/2019	2019/2020	2020/2021	Total
Administration and estates	215 (30)	157 (25)	151 (26)	90 (28)	613 (27)
Healthcare assistants and other support workers	139 (20)	135 (21)	132 (23)	92 (28)	498 (22)
Medical and dental	13 (2)	21 (3)	30 (5)	7 (2)	71 (3)
Nursing, midwifery and health visiting	251 (36)	220 (35)	180 (32)	101 (31)	752 (34)
Scientific, therapeutic and technical	42 (6)	62 (10)	48 (8)	21 (7)	173 (8)
Other	16 (2)	15 (2)	17 (3)	11 (3)	59 (3)
Missing	31 (4)	19 (3)	13 (2)	1 (0)	64 (3)
Total	707 (100)	629 (100)	571 (100)	323 (100)	2230 (100)

Percentages by year in parentheses

#### Dependent variable

Reasons for non-acceptance were analysed and coded using an extended version of the categorisation method used by Hollmeyer et al. [14] (Table 3). Categories 1–9 were the same as Hollmeyer et al. and categories 10 (Other) and 999 (Missing) were added to fully capture responses given by the staff members from the ABUHB. If more than one reason was given, the first reason stated was coded.

**Table 3:** Categorisation of reasons for vaccine non-acceptance

Code	Category	Examples of identified reasons
1	Lack of concern	“Influenza is not a serious disease”
2	Lack of perception of own risk	“I don’t work with patients”, “don’t feel I need it”, “no history of flu”, “Never had it”, “Not needed if you have a diet that includes enough fruit, water and veg”, “I am young and healthy and don’t fell I need this”, “Fit and Well, never been sick, Not had a cough/cold in 10 years", “don’t need it”, “healthy enough”, “Prefer to use methods to improve immune system naturally”, “I don’t need it”
3	Doubts about vaccine efficacy	“The vaccine does not work”, “empirical data”, “not convinced by the evidence”, “pointless”
4	Fear of adverse reactions	“Fear of side effects”, “poorly after the last one”, “I don’t feel there has been enough research into the vaccine”, “heard bad stories”, “very sick previously
5	Self-perceived contra-indications	“Allergy”, “currently have a cold”, “Dr told never to have it again”, “currently have a chest infection”, “advised not to have it”, “Medical advice”, “strict vegan”, “pregnant”, “On COVID trial”
6	Dislike of injections	“Needle phobia”, “Too scared”, “phobia”, “it could hurt”, “needle phobic”
7	Avoidance of medications	“I avoid medications”, “does not believe in it”, “Not in agreement with the immunisation”, “I’m a conspiracy theorist”, “don’t think they are good for you”, “Dislike the use of chemicals”, “against my beliefs”, “Prefer to adopt alternative forms of protection”, “take Vitamin D”
8	Lack of availability	“Not offered vaccine”
9	Inconvenient delivery	“Not [at location] when clinic was arranged”, “timing not convenient”, “have with GP”
10	Other	“No”, sorry none I can think of”, “don’t want it”, “I don’t want it”, “Don’t want it”, “Don't wish to receive it”, “Personal beliefs”, “Personal choice”, “Prefer not to”, “My decision”, “Freedom of choice”, “no”, “no comment”, “unwilling to share a reason”, “no particular reason”,
999	No response	“No response given”, “unknown”, “none”, *blank space*”

Responses were categorised using categories 1–9, adapted from Hollmeyer et al. [14]. Categories 10 and 999 were added to capture additional reasons given by staff at ABUHB

Coding of declination text was completed by two independent coders. There was initial discussion about how certain types of responses should be coded but subsequently, agreement was 98% (not including 999 responses). Disagreements were then resolved through discussion.

### Analysis

The primary analysis consisted of first classifying responses according to Table 3 and then comparing counts across time and staff groups. Counts of responses in each category (Table 3) were subjected to a χ^2^ analysis to determine whether there were differences in proportions of reasons across years or staff groups respectively. A *p*-value of *p* < 0.05 was taken to be significant.

Contingency tables were tested to ensure that they were consistent with assumptions of the χ^2^ test i.e. cell expected values should not be less than 1 nor should there be greater than 20% of cells with an expected value of less than 5 [32].

Pearson standardized residuals (SRs), i.e. *z-*scores, were used to establish which cells in the contingency tables differed significantly from their expected values. SRs greater than 1.96 were considered to be significant at the 0.05 level.

All analyses were completed using IBM SPSS 27.

A post hoc subcategory analysis was employed to identify occurrences of particular hypothesis-related expressions within *Fear of adverse reactions* and *Lack of perception of own risk.* More detail is shown in the Results section.

## Results

### Analysis across years

#### Data cleaning

The categories *Lack of concern* and *Lack of availability* contained mostly zero counts (Table 4). To avoid low expected values in the contingency table, we removed these categories from the analysis. The resulting table consisted of 9 response categories across 4 years. The minimum expected value was 4, and 2.8% (1 cell) had an expected count of less than 5.

**Table 4:** Number of declination reasons by year

Reason category	2017/2018	2018/2019	2019/2020	2020/2021	Total
Lack of concern	0 (0)	0 (0)	1 (0)	0 (0)	1 (0)
Lack of perception of own risk	72 (10)	41 (7)	42 (7)	15 (5)	170 (8)
Doubts about vaccine efficacy	17 (2)	5 (1)	7 (1)	7 (2)	36 (2)
Fear of adverse reactions	123 (17)	69 (11)	47 (8)	42 (13)	281 (13)
Self-perceived contra-indications	40 (6)	36 (6)	27 (5)	18 (6)	121 (5)
Dislike of injections	33 (5)	16 (3)	9 (2)	8 (2)	66 (3)
Avoidance of medications	10 (1)	9 (1)	6 (1)	6 (2)	31 (1)
Lack of availability	0 (0)	0 (0)	0 (0)	1 (0)	1 (0)
Inconvenient delivery	14 (2)	10 (2)	19 (3)	10 (3)	53 (2)
Other recorded response	92 (13)	50 (8)	43 (8)	10 (3)	195 (9)
Missing	306 (43)	393 (62)	370 (65)	206 (64)	1275 (57)
Total	707 (100)	629 (100)	571 (100)	323 (100)	2230 (100)

Percentages by year shown in parentheses

#### Analysis

**Fig. 1:**
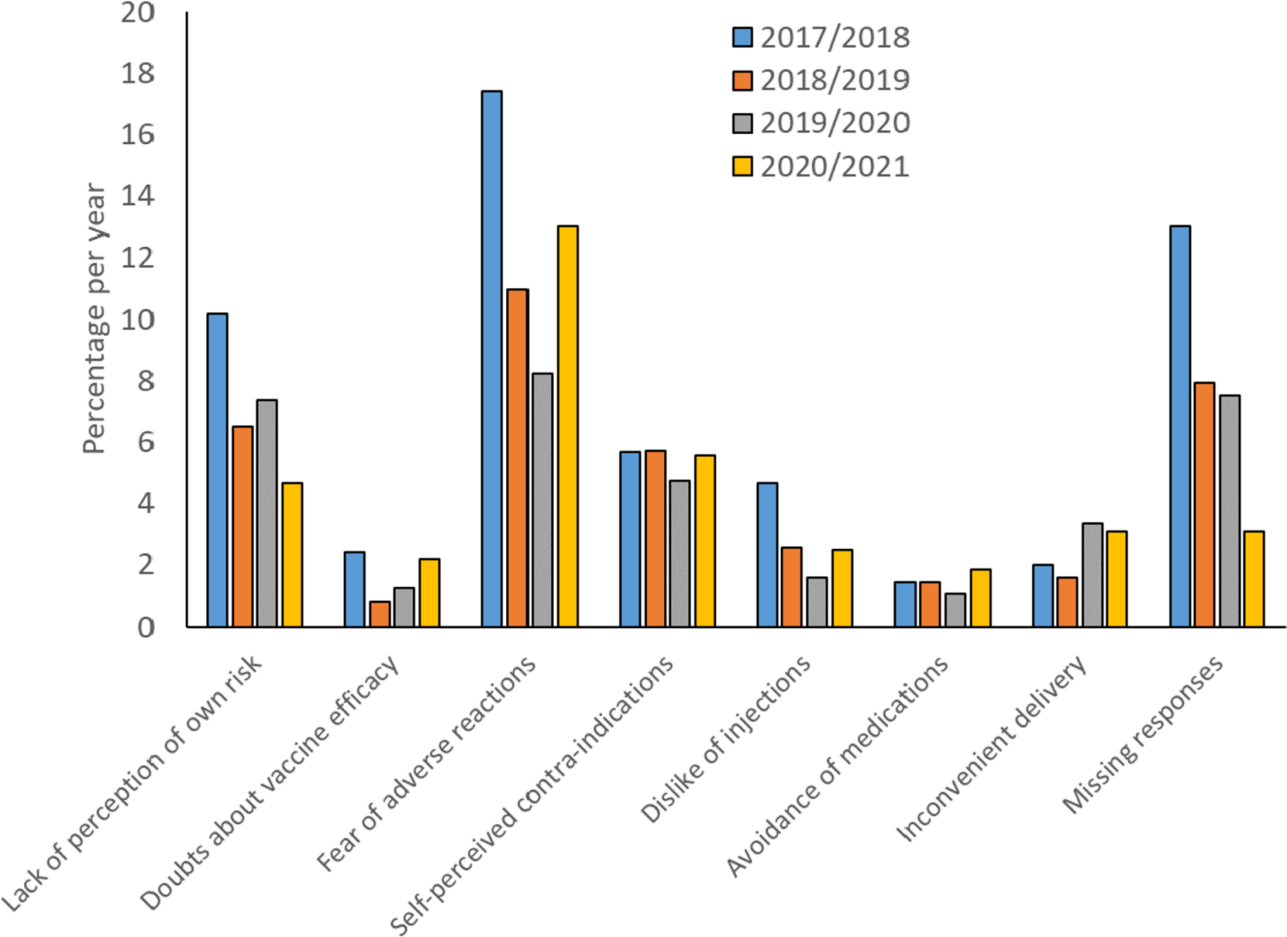
Percentage of declination responses by category and year (see Table 4). 2020/2021 is post-COVID-19 arrival. Note that *Missing* responses (57%), *Lack of concern* (0%), and *Lack of availability* (0%) are not plotted for presentation reasons. *Fear of adverse reactions* is the highest category for each year

There were clear patterns in responses (Fig. 1). *Fear of adverse reactions* was the most cited category, while there were almost no *Lack of concern* or *Lack of availability* responses. *Lack of perception of own risk* and *Other recorded* responses dropped across years, with the lowest percentage seen post-COVID, 2020/2021. However, 57% of declination forms contained no response (Table 4).

A χ^2^ test on the 9 (response category) × 4 (year) contingency table revealed differences across years in the frequencies of response assigned to different categories, χ^2^(24) = 123.10, *p* < 0.001. As measured by standardized residuals, there were more counts than expected of *Dislike of injections* in 2017/2018, SR = 2.6, and fewer in 2019/2020 SR = -1.9; more counts of *Fear of adverse reactions* in 2017/2018, SR = 3.5, and fewer in 2019/2020 SR = -3.0; more *Other recorded* reasons in 2017/2018, SR = 3.8, and fewer in 2020/2021, SR = -3.4, more *Lack of perception of own risk* in 2017/2018, SR = 2.6, and fewer in 2020/2021, SR = -2.0; and more *Missing* responses in 2018/2019, SR = 1.8, and 2019/2020, SR = 2.4, and fewer in 2017/2018, SR = -4.9. No other SRs were greater than 1.96.

Of particular interest were comparisons between pre- and post-COVID-19 arrival. We therefore combined 2017/2018, 2018/2019, and 2019/2020, and compared them against 2020/2021. There were significant differences observed between pre-and post COVID-19 pandemic, χ^2^(8) = 28.93, *p* = 0.002. However, differences were now restricted to two categories: Reductions in *Lack of perception of own risk*, SR = -2.0, and *Other recorded responses*, SR = -3.4. No other SRs were greater than 1.96.

### Subcategory analysis

#### Fear of adverse reactions

We tested whether the adverse reactions participants feared were consistent with known reactions to the vaccine, or to misconceptions.

The two adverse reactions that have been associated with the influenza vaccine [33] are a small increased risk of fever, and muscle ache (myalgia). There was only one instance of myalgia (out of 281) in the adverse reactions responses, “It paralysed my arm for two days”. There were no other occurrence of “arm”, “muscle”, “ache”, or “pain” (except in an unrelated context). Thus myalgia was not a concern for responders. Fever was more difficult to assess. There were no instances of “fever”, “temperature”, “high”, or “thermometer”. There was therefore no evidence of fever specific fears. However, it is doubtful responders would articulate so clearly their concerns even if fever were an issue. Conversely, responses frequently mentioned general illness post vaccine: 162 (out of 281) responses contained “ill” (e.g., “illness”), “well” (e.g., “didn’t feel well”, “unwell”), or “poorly”. This is consistent with the experience of fever and its associated symptoms, although it is also consistent with general placebo malaise.

We also tested whether responders were concerned that the vaccine caused influenza. However, we found no responses that explicitly stated that the vaccine caused influenza, and only 5 that implied causality e.g., “I had bad flu and cough after 2 flu jabs so I am trying not to have it this year.”

#### Lack of perception of own risk

We wanted to find out in more detail why decliners perceived themselves to be of low risk.

There were 63 responses (out of 168) that contained “never had jab” or similar reference to not having had the vaccine before. There were 43 responses that contained “never had flu” or similar reference to not having had influenza. These co-occurred 11 times. These numbers suggest that a high proportion of decliners believed that because they had never had influenza, they did not need the vaccine.

Many responses (43) referred simply to the absence of “need” e.g., “Feel I don’t need it” without giving further explanation.

### Analysis across staff groups

#### Data cleaning

All four years were pooled. Participants who gave no staff group (*N* = 64) were removed because they provided no information about the association between staff groups and declination reason. To avoid low cell counts for the χ^2^ test, *Lack of concern* (*N* = 1) and *Lack of availability* (*N* = 4) response categories were removed, as were *Other* staff category (*N* = 59). The analysis was therefore based on a 9 (Declination category) by 5 (Staff group). The minimum expected cell count was 1, and 18% (8) cells had an expected count of less than 5.

#### Analysis

In each staff group, the most cited declination reason was *Fear of adverse reactions* (Table 5). However, there were also differences in the distribution of responses among staff groups, χ^2^(32) = 110.49, *p* < 0.001. For *Doubts about vaccine efficacy*, Medical and dental staff had a significantly higher count than expected, SR = 4.7, and Healthcare assistants and other support staff had a significantly lower count, -2.0. *For Inconvenient delivery*, Medical and dental staff had significantly higher counts, SR = 5.5. For *Self-perceived contra-indications*, Medical and dental staff had significantly higher counts, SR = 2.1, and Healthcare assistants and other support staff had significantly lower counts, SR = -3.0. Finally, Medical and dental staff had fewer counts of Unrecorded responses, SR = -2.0. There were no significant differences across staff groups for *Avoidance of medications*, *Dislike of injections*, *Lack of perception of own risk*, or *Other recorded* responses, all SR’s < 1.96.

**Table 5:** Number of declination forms as a function of category and staff group

	Not provided	Administration and estates	Healthcare assistants and other support workers	Medical and dental	Nursing, midwifery and health visiting	Other	Scientific, therapeutic and technical	Total
Lack of concern	0 (0)	4 (1)	0 (0)	0 (0)	0 (0)	0 (0)	0 (0)	4 (0)
Lack of perception of own risk	4 (6)	51 (8)	32 (6)	5 (7)	53 (7)	7 (12)	12 (7)	164 (7)
Doubts about vaccine efficacy	2 (3)	5 (1)	2 (0)	6 (8)	15 (2)	2 (3)	4 (2)	36 (2)
Fear of adverse reactions	4 (6)	66 (11)	80 (16)	10 (14)	101 (13)	4 (7)	17 (10)	282 (13)
Self-perceived contra-indications	4 (6)	33 (5)	12 (2)	8 (11)	52 (7)	3 (5)	11 (6)	123 (6)
Dislike of injections	3 (5)	24 (4)	17 (3)	2 (3)	15 (2)	0 (0)	5 (3)	66 (3)
Avoidance of medications	0 (0)	10 (2)	4 (1)	0 (0)	12 (2)	2 (3)	3 (2)	31 (1)
Lack of availability	0 (0)	1 (0)	0 (0)	0 (0)	0 (0)	0 (0)	0 (0)	1 (0)
Inconvenient delivery	0 (0)	16 (3)	6 (1)	9 (13)	12 (2)	2 (3)	8 (5)	53 (2)
Other recorded response	11 (17)	58 (9)	40 (8)	3 (4)	60 (8)	6 (10)	17 (10)	195 (9)
Missing	36 (56)	345 (56)	305 (61)	28 (39)	432 (57)	33 (56)	96 (55)	1275 (57)
Total	64 (100)	613 (100)	498 (100)	71 (100)	752 (100)	59 (100)	173 (100)	2230 (100)

Percentages by staff group in parentheses

The data pre- and post-COVID-19 were compared for individual staff groups. However, Nursing, midwifery, and health visiting was the only group that had sufficient data in the post-COVID-19 year for the analysis to be valid, and showed no significant differences, χ^2^(8) = 11.61, *p* = 0.17. Other staff groups had over 33% expected cell counts of less than 5.

## General discussion

We analysed responses given by HCW when declining the influenza vaccination. The analysis used four years of data, pre- and post- arrival of the COVID-19 pandemic, and included six categories of staff. There were stable patterns of choices across years and staff groups, and also differences. This demonstrates that declination data can be an informative data collection tool, not only an intervention to improve vaccination uptake [24, 26]. We now discuss the specifics of our findings.

### Reasons for declination

We found that *Fear of adverse reactions* was the primary reason (30%) for refusal, consistent with other studies [9, 10, 14, 15, 30, 34]. Moreover, our study shows that these concerns were sufficiently broad that they were held for every staff group, and for four consecutive years in the same health environment, including post-COVID arrival.

The majority of responses in *Fear of adverse reactions* described fears around feeling generally ill after the vaccine e.g., “often became unwell afterwards.” Other studies [9, 10, 14, 15, 30, 34] have found similar responses. For example, Ferragut et al. [10] found that 37 out of 94 HCW who did not accept the vaccine cited “I got sick after the vaccine” as the reason. However, we found no evidence of specific concerns around recognised side-effects of the vaccine, in particular myalgia or fever, although feeling ill is of course consistent with fever. The absence of specifics amongst HCW suggests that their concerns were based on erroneous causal inferences between vaccine and illness, or placebo effects rather than genuine side effects of the vaccine.

There was one noticeable difference between adverse reaction responses in this study compared to previous studies. Here, we found no evidence of the widely reported myth that the vaccine gives the recipient influenza [11, 13, 20, 30, 35–37]. This contrast could be because of different levels of influenza knowledge across samples used in studies (ABUHB had vaccine education campaigns throughout the period under study). Another possibility is that difference is due to variation in data collection techniques. ABUHB used free-text responses in the declination forms whereas other studies, e.g. Ribner et al. [30], used pre-determined response options that explicitly offered vaccines causing influenza as a possibility.

*Lack of perception of own risk* was also a commonly reported category, consistent with most previous studies [14, 38]. Moreover, our data presents a detailed breakdown of how HCW were perceiving their risks: a third declared that they had never had influenza, a third declared that they had never had the vaccine, and most of the remainder declared simply that they had no need for it.

Conversely, we found almost no responses that were classified as *Lack of concern* about influenza. This contrasts with the results of Hollmeyer et al. [14] who found that *Lack of concern* was the second most cited declination reason (see also subsequent studies [39, 40]). This result might reflect a change across time in perceptions of influenza seriousness due to HCW information campaigns. Intuitively, media attention from pandemics in the intervening years, such as H1N1 or COVID-19, might explain the difference between our findings and Hollmeyer et al. However, studies post-H1N1 demonstrated *Lack of concern* as a factor [39, 40], and there were few *Lack of concern* reasons in the pre-COVID-19 years in our data. It therefore seems unlikely that media attention from other pandemics explains the absence of *Lack of concern* responses here.

Another explanation is that different methods of data collection were used across studies: when presented with the opportunity to receive the vaccine in a healthcare setting by a healthcare professional, the seriousness of influenza and the importance of receiving the vaccine could be more salient than when presented in a questionnaire or a focus group.

We also observed few *Doubts about vaccine efficacy* (4%). This contrasts with older studies [14, 15, 17, 34, 41, 42]. For example, Petek and Kamnik-Jug [34] found vaccine efficacy cited in 37% of refusals. As with *Lack of concern*, it is possible that education campaigns on the effectiveness of influenza vaccines are now starting to be integrated into HCW knowledge. Possibly this is also due to different methods of data collection, but recently Ferragut et al. [10] found no questionnaire responses that gave “It does not work” as a reason, consistent with an education explanation.

### Variation across years

The distribution of responses was similar across years. However, there were some differences pre and post-COVID-19 arrival: proportionally fewer *Lack of perception of own risk* responses (e.g. “don’t feel I need it”) than pre-COVID-19, and fewer *Other* responses. This result therefore reflects a change towards a more positive vaccine attitude. Possible reasons for the relative drop in *Lack of perception of own risk* responses post-COVID-19 were that HCW were reminded of their own fallibility from COVID-19, and that there was media discussion about the dangers of influenza during coverage of COVID-19.

More positive attitudes towards influenza vaccination post-COVID-19 are consistent with most previous literature [20, 21, 23, 42]. For example, Wang et al. [23] found that more nurses changed from negative vaccine attitudes pre-COVID-19 to positive attitudes post-COVID-19, than those changed from positive attitudes pre-COVID-19 to negative attitudes post-COVID-19. However, Siani & Tranter [22] found that respondents were less confident in general vaccines post-COVID-19 than pre-COVID-19, somewhat at odds with our findings. The difference could be explained by the use of different respondents across studies, HCW *vs* general population, and the difference in type of vaccination, influenza vaccination *vs* general vaccination.

### Variation across staff groups

For all staff groups, *Fear of adverse reactions* was the most commonly cited reason. We can find few previous studies that strata refusal reasons by staff groups but those that do [10, 30] also show *Fear of adverse reactions* to be the most common reason irrespective of staff group.

There were two groups that showed different distributions to the others. First, Medical and dental staff were relatively more concerned with inconvenient delivery, vaccine efficacy, and contra-indications, and less likely to have missing responses. Such differences might suggest that targeted interventions, such as educational campaigns that assume a high level of medical knowledge, are necessary to address the concerns of clinicians, especially in countries where vaccination rates for clinicians are low (e.g. Slovenia, 13% vaccination rate for Physicians [34]).

Second, Healthcare assistants and other support workers exhibited some significant distribution differences. These were differences in magnitude, however, while overall the pattern of reasons was similar to other staff groups (excluding Medical and dental).

### Myths and misinformation

Low influenza vaccination uptake is often attributed to misconceptions around the vaccine and influenza [14, 38, 43]. For example, Bachtiger et al. [20] (Abstract) state that “164 [decliners out of] 543 (30.2%) gave reasons based on misinformation”, and Ferragut et al. [10] conclude that, “Myths, fears and misinformation are the main barriers for refusal of the flu vaccine.”

Our data support the view that misconceptions play a role but we note that the misconceptions present in our sample were not straightforward factual errors. Specifically, we saw little evidence that decliners believed the vaccine caused influenza, nor that they believed influenza wasn’t a concern. Likewise, the adverse reactions feared by decliners were consistent with known side-effects of the vaccine e.g. “feeling ill” is consistent with the elevated risk of fever associated with the vaccine [33]. Rather than being factual errors, the misconceptions were those of reasoning and risk perception. For example, many responses suggested that HCW thought that because they had not had influenza before (and not had the vaccine), they had a low risk of getting influenza; others believed that because they had never had the vaccine before, there was no need to have it now. Similarly, the prevalence of fears about being ill after the vaccine suggest over-estimation of the risk of side effects (Demichelli et al., [33], estimate that inactivated vaccines increase the risk of fever by around 0.8% relative to control). This view is consistent with questionnaire findings showing risk perception to be a significant predictor of whether HCW accept the vaccine [44, 45].

Attributing low uptake to risk perception rather than more general misconception has implications for educational campaigns targeting HCW. Although it is important to reinforce the basics of the influenza vaccine (e.g., that the vaccine is inactive), focus could now be shifted towards ensuring HCW have a more accurate understanding of risks involved in contracting influenza and receiving the vaccine.

### Limitations

The primary limitation of this study is the missing data. There were two manifestations of this. First, many declination forms did not contain a reason for the declination (1275 out of 2230). This could be because immunisers did not ask HCW or because HCW declined to give reasons. Second, only a self-selecting and small proportion of staff who declined the vaccine completed the form, and this number fell across years. Both cases of missing data are problematic because the distribution of declination reasons may be different for the missing data than for the recorded data. Note, however, that even with the missing data, the sample number and distribution across staff groups was far greater than many questionnaire studies (and there is no sampling method free from all biases).

A similar issue is that the sample was not chosen to be representative of HCW in ABUHB or elsewhere with respect to age, gender, or socioeconomic status, factors that are known to effect vaccine uptake [38]. It is therefore possible that participants with different demographics characteristics may have a different distribution of reasons for declination.

### Declination forms

Although the aim of this study was to understand why HCW decline the vaccine, the study also speaks to the effectiveness and implementation of declination forms as an approach to improving uptake. First, vaccine uptake in the year declination forms were introduced, 2017/2018, was only modestly higher than before they were introduced, 2016/2017: 58.0% *vs* 52.1% (Table 1), and the increase should be seen as part of a general trend for increasing uptake in the years considered in this study. This contrasts with some large effects reported in the literature. For example, LaVela et al. [24] report 54% pre-implementation and 77% post-implementation of declination forms, and Lytras et al. [25] conclude that declination policies had the largest independent effect of interventions short of mandating vaccination. However, Bell et al. [27], in their review of opt-out polices in England under the NHS, report more modest findings, consistent with our own data. Clearly, declination forms do not necessarily improve uptake, much depends on the environment and other changes taking place in parallel to their introduction.

The second point is that consistent implementation of the declination forms at ABUHB was difficult. In the first year they were introduced, 2017/2018, there were only 707 completed declination forms, yet ~ 4000 HCW were unvaccinated (Table 1), and the number of completed declination forms dropped further in later years (without a proportional increase in vaccination numbers). The absence of declination forms for many staff suggests that there was minimal managerial emphasis on collecting declination forms and/or immunisers did not have the resources to administer them (possibly due to the arrival of COVID-19). This might explain the relatively low increase in uptake compared to other studies.

A third point relates to how decliners were engaged by immunisers. Immunisers were instructed to discuss with decliners why they refused the vaccine and to offer the vaccine again after discussion. The data illustrates that 23 decliners changed their mind after this intervention. While this appears to be a very low number (23/2230), it is not clear how many immunisers engaged with the decliners nor how they did so. The effects of engaging with the decliners may therefore be more powerful than it appears and warrants further research towards making it an effective intervention.

## Conclusion

This study shows how data collected from declination forms can identify reasons why HCW choose to decline the influenza vaccine. Declination data has the advantage that it is collected in vivo so is not subject to retrospective or pre-emptive reasoning. Our findings demonstrate that *Fear of adverse effects*, *Lack of perception of own risk*, and *Self-perceived contra-indications* are the primary declination reasons across all years and all staff groups. However, rather than being due to factual misconceptions, the data suggest the underlying problem to be risk perception: HCW underestimate the risks associated with influenza and overestimate the risks of adverse reactions. We have also shown that *Lack of concern* for the importance of influenza is not a factor in declinations, and that there is a change in attitudes towards perception of risk associated with influenza post-COVID-19.

## Data Availability

Raw data is publicly available at https://osf.io/734tg/?view_only=77213145821449cd9008980ef383b243
